# Myelin dysfunction in aging and brain disorders: mechanisms and therapeutic opportunities

**DOI:** 10.1186/s13024-025-00861-w

**Published:** 2025-06-15

**Authors:** Zhihai Huang, Yulan Zhang, Peibin Zou, Xuemei Zong, Quanguang Zhang

**Affiliations:** 1 Institute for Cerebrovascular and Neuroregeneration Research (ICNR), Department of Neurology, Louisiana State University Health Sciences Center, 1501 Kings Highway, Shreveport, LA 71103 USA; 2 Department of Pharmacology, Toxicology, and Neuroscience, Louisiana State University Health Sciences Center, 1501 Kings Highway, Shreveport, LA 71103 USA

**Keywords:** Myelin, Adaptive myelination, Aging, Brain disorders, Neurodegenerative disease, Remyelination therapy

## Abstract

Myelin is a multilamellar membrane that surrounds axons in the vertebrate nervous system. Properly functioning myelin is essential for the rapid conduction of nerve impulses, and it metabolically supports axonal integrity. Emerging evidence indicates that myelin is also involved in various aspects of cognition, with adaptive myelination playing a critical role in memory consolidation and motor learning. However, these physiological processes can be disrupted in various diseases. Understanding the mechanisms underlying myelin pathology is therefore essential for the development of targeted therapies for associated medical conditions. This review provides a comprehensive overview of the role of myelin in neural function, with a particular focus on adaptive myelination in cognition. We also highlight myelin dysfunction and the underlying mechanisms in the aging brain, as well as in diverse brain disorders and neurological conditions, including neurodegenerative diseases, psychiatric conditions, brain injuries, chemotherapy-related cognitive impairment, and neurological symptoms associated with COVID-19. Furthermore, we discuss the therapeutic potential of recently identified pro-myelinating compounds in aging-associated cognitive decline and brain disorders, as well as the future of remyelination therapies. Current evidence suggests that restoring functional myelin may serve as a therapeutic strategy for various medical conditions associated with myelin dysfunction.

## Background

Myelin is a multilamellar membrane that wraps neuronal axons in the nervous system. As an evolutionary adaptation, myelinated axons ensure the rapid propagation of nerve impulses. Myelin also supports axonal integrity and plays a role in maintaining higher cognitive functions [1, 2]. Emerging evidence suggests that well-functioning myelin, along with myelination—the formation of new myelin by myelin-forming glial cells—contributes to memory consolidation and various aspects of cognitive functioning [3–6]. This insight has sparked a growing interest in investigating the contribution of myelin pathology to brain disorders marked by myelin dysfunction.

Accumulating evidence from cell culture studies, preclinical animal models, postmortem analyses, and neuroimaging studies suggests that myelin dysfunction is involved in aging-associated neurological deficits and a wide range of brain disorders. These disorders include Alzheimer’s disease (AD) [7–9], multiple sclerosis (MS) [10–12], depression [12–14], stroke [15–17], traumatic brain injury (TBI) [18–20], schizophrenia [21–23], as well as other conditions ranging from hypoxic brain injury [24, 25] to COVID-associated neurological symptoms [26]. Although these conditions arise from distinct mechanisms, they share the common feature of disrupted myelin integrity, which may underlie certain observed neurological deficits.

In this review, we provide an overview of the current understanding of the functional significance of myelin and highlight key pro-myelinating compounds. We focus in particular on myelin dysfunction and its underlying mechanisms in the aging brain and various neurological conditions. The potential of remyelination therapy for aging and brain disorders is also discussed. Converging evidence suggests that myelin may represent a promising therapeutic target for these conditions.

### Myelin in the central nervous system

**Fig. 1:**
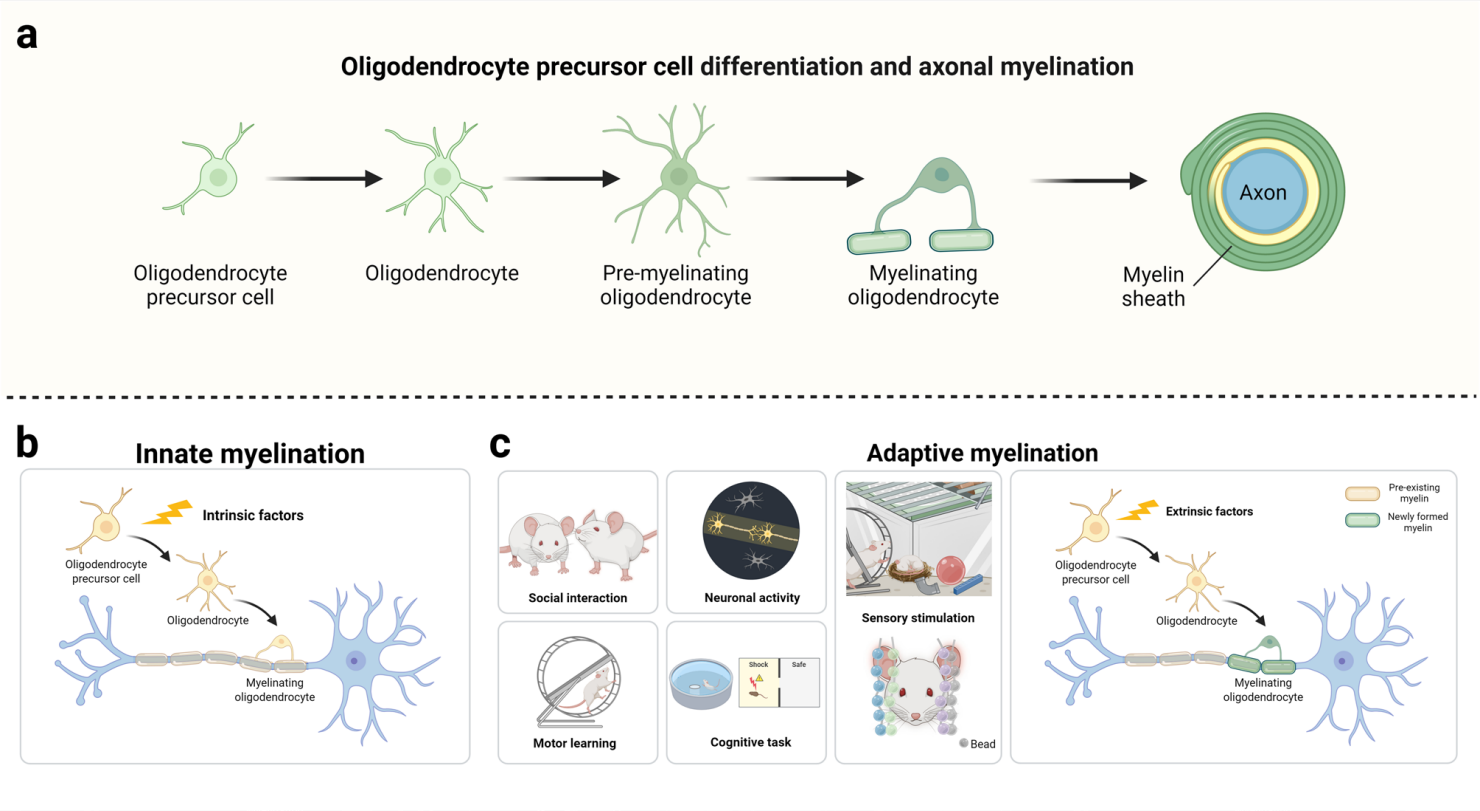
Oligodendrocyte lineage cell development and myelination. **a** Schematic depiction of oligodendrocyte precursor cell differentiation and axonal myelination. **b** Schematic representation of innate and adaptive myelination. Innate and adaptive myelination persist throughout the lifespan and are influenced by intrinsic and extrinsic factors, respectively

Myelin is a unique evolutionary adaptation in vertebrates. This multilamellar membrane, produced by oligodendrocytes in the central nervous system (CNS) and Schwann cells in the peripheral nervous system (PNS), wraps around axons to form an insulating layer. In the CNS, oligodendrocytes arise from oligodendrocyte precursor cells (OPCs), which originate in the ventricular zones and subsequently migrate throughout the CNS [27, 28]. Upon exiting the cell cycle, OPCs terminally differentiate into oligodendrocytes. These newly differentiated oligodendrocytes progress through developmental stages—from premyelinating to myelinating oligodendrocytes—ultimately wrapping axons to form the myelin sheath (Fig. 1).

In mammals, while most myelin is formed early in life through intrinsic mechanisms, extrinsic factors like experience and environmental stimuli also shape myelination [29] (Fig. 1). This gives rise to two categories of myelination: innate myelination and adaptive myelination. Both intrinsic and extrinsic factors influence myelination throughout the lifespan [2, 30]. As a complex process, myelination is regulated by diverse signal transduction pathways, which have been elegantly reviewed elsewhere [31, 32].

Adaptive myelination is recognized as one of the critical mechanisms underlying neuroplasticity, driven by various extrinsic cues and neuronal activity during both development and adulthood. Animals exposed to enriched environments exhibit higher myelin levels in the brain [33–35], and sensory enrichment increases oligodendrogenesis and oligodendrocyte integration in the somatosensory cortex of middle-aged animals [36]. Moreover, different learning tasks trigger increased differentiation of OPCs and enhanced myelination [4, 5, 37]. Conversely, early postnatal sensory deprivation disrupts axonal myelination and leads to behavioral changes in mice [38, 39]. It has been shown that social isolation during a critical post-weaning period results in lower myelin levels and thinner myelin in the medial prefrontal cortex (mPFC) compared to regularly housed mice, a deficit that cannot be recovered by later social reintroduction [40]. However, social reintroduction can reverse myelin abnormalities when isolation occurs beyond this critical period [41]. Additionally, pharmacological enhancement of myelination has been shown to reverse hypomyelination and social avoidance behavior in socially isolated adult mice [42].

In the past few decades, studies have demonstrated that changes in neuronal activity also regulate myelination. One such study shows that blocking or enhancing neuronal firing with different toxins inhibits or enhances myelination, respectively [43]. This study was the first to reveal a potential link between neuronal electrical activity and myelination. The notion that neuronal activity regulates myelination is further supported by emerging evidence. For example, optogenetic stimulation of the premotor cortex in awake mice has been shown to increase OPC differentiation, promote oligodendrogenesis, and enhance myelination in the premotor cortex and subcortical white matter [44]. Conversely, blocking neuronal activity by tetrodotoxin in demyelinated lesions reduces remyelination, as demonstrated in a toxin-induced demyelination model [45]. Similar findings have been reported in studies using chemogenetic or pharmacogenetic strategies to stimulate neuronal activity [46, 47].

### Functional significance of Myelin in the CNS

**Fig. 2:**
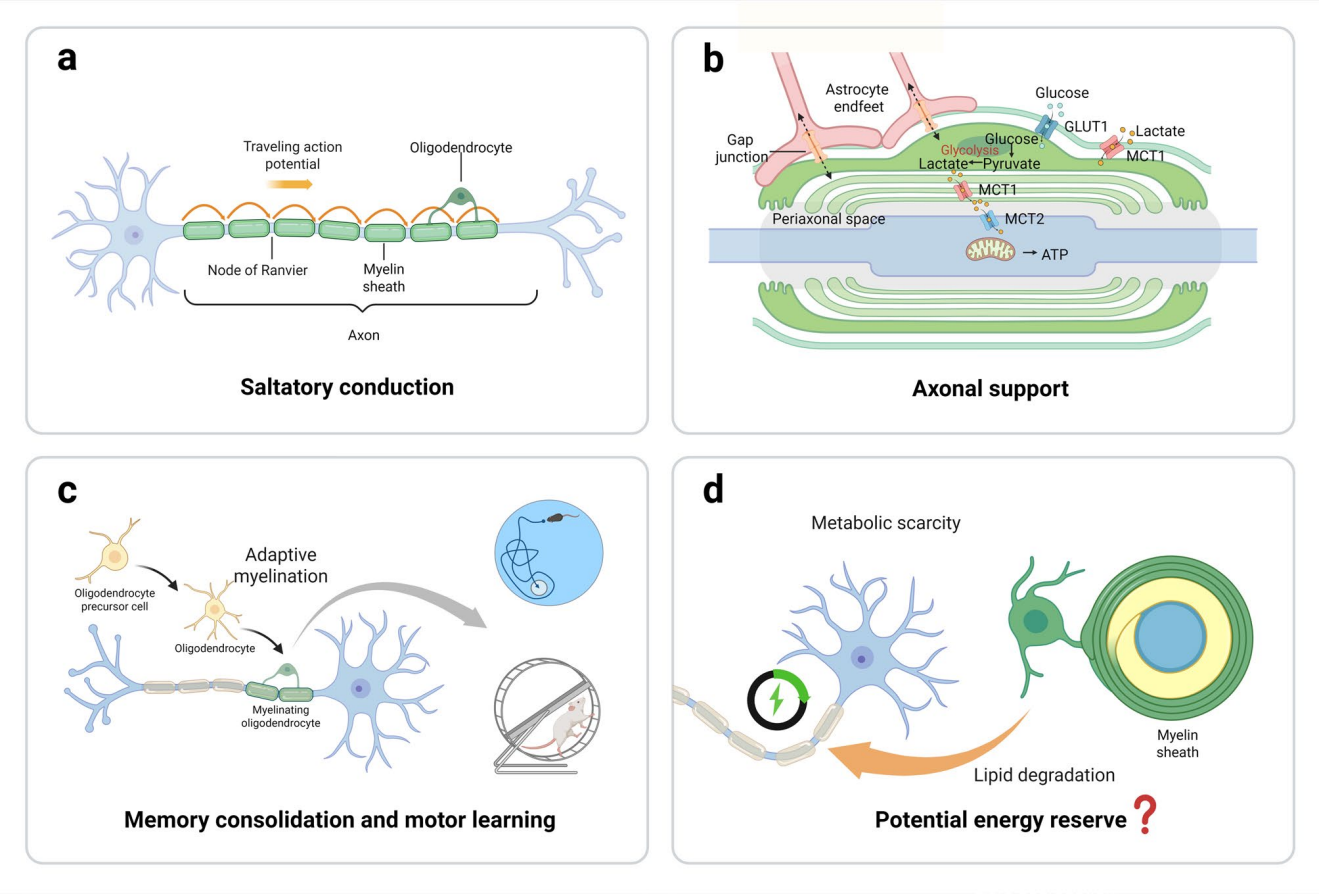
Functional significances of myelin in the CNS. **a** Saltatory conduction allows for the fast propagation of action potentials along the axon. **b** Myelin provides metabolic support to axons, which is crucial for maintaining axonal integrity under normal physiological conditions. **c** Learning and motor skill acquisition lead to new myelin formation. This adaptive myelination is crucial for memory consolidation and the acquisition of motor skills. **d** Under energy scarcity, myelinating oligodendrocytes may undergo lipid degradation to fuel neural cells

While traditionally considered primarily as an insulating multi-lamellar structure enabling rapid nerve transmission, myelin is now known to play multiple physiological roles. In the following section, we review the functional significance of myelin in the CNS, including (1) action potential propagation and axonal metabolic support, (2) memory consolidation and motor learning, and (3) its potential function as an energy reserve (Fig. 2).

### Saltatory conduction and axonal support

As an insulating layer around axons, one of the most critical functions of myelin is to facilitate the propagation of action potentials. Small unmyelinated gaps called Nodes of Ranvier separate adjacent myelin sheaths, forming sites where voltage-gated sodium channels cluster at high density. The myelin sheath restricts action potentials to these highly specialized gaps between myelinated segments, allowing action potentials to “jump” rapidly from node to node, enabling conduction speeds 50- to 100-fold faster than in unmyelinated axons [2, 48]. Furthermore, by reducing transverse capacitance and increasing transverse resistance of the internodal axonal membrane, myelin minimizes ion leakage across the axonal membrane and reduces axonal energy consumption [49, 50].

Apart from its role as an insulating membrane structure, the myelin sheath also provides metabolic support to axons and is crucial for their long-term integrity. Indeed, due to the presence of the myelin sheath, myelinated axons are shielded from the nutrient-rich extracellular environment and rely on oligodendrocyte lineage cells (OLs) and myelin to meet their metabolic demands [50]. The metabolic support myelin provides to axons relies on the shuttling of energy metabolites between the inner myelin membrane and the axon. Oligodendrocytes uptake glucose through glucose transporter 1 (GLUT1), metabolizing it into lactate and pyruvate. Gap junctions connecting astrocytes and oligodendrocytes also facilitate the transfer of energy metabolites. The glycolysis products, pyruvate and lactate, are transported to the internodal periaxonal space and subsequently absorbed by the axon via monocarboxylate transporters (MCT1 and MCT2) [1, 51, 52]. Oligodendrocyte-specific knockdown of MCT1 induces axonal injury in both organotypic slices and animal models [53]. However, supplementing _L_-lactate to the culture media rescues neurodegeneration caused by MCT1 knockdown and glucose deprivation in organotypic slices [53]. MCT1 in oligodendrocytes not only functions as a cotransporter of energy metabolites across the membrane but also facilitates the direct uptake of extracellular lactate into oligodendrocytes, a process that is critical for the development and myelination of oligodendrocytes. Studies have demonstrated that under low-glucose conditions, the number of oligodendrocytes and the extent of myelination decrease in cultured slices of mouse cerebral cortex, a deficit that can be rescued by _L_-lactate supplementation [54]. Recent findings reveal that the deletion of MCT1 in oligodendrocytes leads to late-onset hypomyelination and axonal degeneration, further underscoring the critical role of myelin in the metabolic support of axons [55]. Additionally, high-resolution electron microscopy has revealed a cytoplasm-rich “myelinic channel system” within myelin sheaths, maintained by the interaction between CNP and F-actin [52, 56]. It has been suggested that this system may be involved in metabolic support for axons, although its exact function remains unclear [1].

While healthy myelin provides essential metabolic support to axons, dysfunctional OLs or myelin—particularly when it remains wrapped around the axons—may become detrimental to axonal health. This concept is supported by evidence showing that fully demyelinated axons appear preserved in a mouse model of demyelination, whereas distal axonal segments with residual myelin are more affected by swelling, which is associated with immune cell-mediated active inflammation [57]. Similarly, mice with dysfunctional myelin caused by genetic ablation of key myelin-associated proteins exhibit widespread axonal swelling and degeneration, whereas these pathologies are absent in shiverer mice and Long Evans shaker rats—strains lacking compact myelin [58–60]. Consistent with this, a recent study has shown that axons ensheathed by perturbed myelin pose axon at the risk of degeneration, driven by CD8^+^ T cell, while the removal of perturbed myelin combats CD8^+^ T cell-driven axon degeneration [61]. In an autoimmune environment, insulation of axons by dysfunctional myelin may be detrimental to axonal health. In a transgenic mouse model with reduced CNS myelination, axonal damage was largely confined to myelinated axons during the early stages of autoimmune disease [62]. Of note, this does not imply that myelin itself inherently puts axons at risk of degeneration. Although dysfunctional myelin could be deleterious, there is currently no evidence that promoting demyelination provides any benefits in pathological conditions. Rather, chronic demyelination may further increase the vulnerability of axons to degeneration. Conversely, the ensheathment of axons with healthy myelin—or the restoration of myelin—remains essential for maintaining axonal function and integrity.

### Adaptive myelination is required for memory consolidation and motor learning

Experiments in mice with myelin deficits reveal that severe hypomyelination leads to an anxious phenotype, abnormal stress responses, and cognitive impairments [3]. These findings suggest that myelin plays a crucial role in higher cognitive functions and that its dysfunction may contribute to cognitive deficits and mental illnesses. Indeed, recent studies have revealed that adaptive myelination is essential for the consolidation of memory and motor learning. In mice, water maze learning results in enhanced oligodendrogenesis and de novo myelination in the cortex and associated white matter tracts [4]. However, deletion of the myelin regulatory factor (Myrf), a transcription factor critical for OPC differentiation, using a tamoxifen-inducible Cre/loxP system, leads to impaired memory consolidation without affecting spatial learning [4]. Similar findings were observed in mice trained on T-maze and radial-arm-maze tasks, which assess spatial working memory [6]. Another study has demonstrated that fear learning increases OPC proliferation as early as 24 h after contextual fear conditioning in the mPFC [5]. Animals subjected to contextual fear conditioning also exhibit increased myelinated axons in the mPFC 30 days after fear learning. Notably, animals unable to generate new myelin show deficits in remote fear memory recall but not in recent fear memory recall [5]. In contrast, pharmacological enhancement of myelination improves remote memory recall and promotes fear generalization, highlighting the important connection between adaptive myelination and memory consolidation [5].

A growing body of studies has revealed that adaptive myelination plays a role in various motor skill learning processes [37, 63–65]. Mice exposed to a “complex wheel” with irregularly spaced rungs gradually learn to run on it, a process accompanied by increased oligodendrogenesis in the sensorimotor cortex [37, 63]. However, blocking OPC differentiation, without affecting preexisting myelin, prevents the mice from acquiring this complex motor skill [37, 63]. This idea is further supported by the observation that mice undergoing forelimb reach training, where they are trained to use their left paw to retrieve food pellets, show myelin remodeling in the motor cortex [64]. Studies using longitudinal two-photon in vivo imaging have revealed that, although the forelimb reach training transiently suppresses oligodendrogenesis, it subsequently increases OPC differentiation, oligodendrogenesis, and myelin remodeling in the motor cortex. Interestingly, in mice with demyelination, while the total number of reach attempts remains unchanged, their success rate in retrieving the pellet following training is significantly reduced [64]. Likewise, mice with myelin deficits exhibit impaired motor learning in a self-initiated lever-pull task, in which they were trained to pull and hold a lever to receive a reward [65]. In a neuroimaging study with healthy young adults, visuomotor skill training with the right arm leads to significantly increased myelin levels in task-dependent brain regions, including the left intraparietal sulcus and left parieto-occipital sulcus, compared to baseline levels [66].

Overall, these studies underline the critical role of adaptive myelination in memory consolidation and various motor skill learning. With these enhanced insights into the functional significance of myelin, it has become a focus of intense investigation in various neurological conditions.

### Myelin as a potential energy reserve

Studies using isolated mouse optic nerves have shown that oligodendroglial fatty acid metabolism sustains axonal function under low-glucose conditions [67]. Indeed, lipids constitute the major component of the myelin sheath, accounting for 70% of its composition. Emerging evidence suggests these myelin lipids may also serve as glial energy reserves during extreme metabolic conditions. Reduced myelin water fraction signal has been observed in white matter areas involved in motor coordination, sensory processing, and emotional integration 2 days after marathon running [68]. This reduction appears to be reversible, with values gradually returning to pre-marathon levels within two months [68].

These findings allow for the hypothesis that myelin may serve as an energy reserve during periods of metabolic scarcity. However, direct evidence for this idea remains limited, and further investigation is needed to determine whether the transient reduction in the signal of white matter myelin water fraction, following prolonged endurance running, represents an adaptive response or a form of metabolic support for brain function. A deeper understanding is also required to clarify the extent to which myelinating oligodendrocytes or myelin lipid degradation can fuel axons—and under what conditions this process occurs. Overall, although this potential physiological role of myelin remains inconclusive, further investigation is expected to provide novel insights into myelin plasticity.

### Identification of pro-myelinating compounds

Since many neurological conditions are characterized by the loss of myelin and axonal integrity, the identification of pro-myelinating compounds may lead to novel therapeutic strategies and has become a major focus of research in the field. High throughput screening has led to the discovery of numerous promising pro-myelinating compounds (Table 1). Among these, benztropine—an FDA-approved medication for Parkinson’s disease—was the first pro-myelinating compound identified using this approach. Benztropine has been shown to promote OPC differentiation and myelination in OPC and neuron co-cultures in vitro and to enhance myelin renewal in various animal models of demyelination in vivo [69]. Its pro-myelinating effects are attributed to M1 and M3 muscarinic acetylcholine receptor antagonism, as co-treatment with muscarinic acetylcholine receptor agonists blocks OPC differentiation induced by benztropine treatment [69]. In addition, using micropillar arrays, a high-throughput screening platform, a cluster of antimuscarinic compounds was identified to promote OPC differentiation and remyelination [70]. Among these bioactive compounds, clemastine fumarate, an FDA-approved antihistamine with antimuscarinic properties, proved to be the most effective in enhancing OPC differentiation and myelination in vitro. Clemastine fumarate administration effectively facilitated axonal remyelination in vivo following local demyelination [70]. Subsequent studies revealed that its effects on OPC differentiation are mediated specifically through antimuscarinic activity at the OPC M1 muscarinic acetylcholine receptor, with no significant involvement of other receptor subtypes [71].

**Table 1:** Summary of identified pro-myelinating compounds

Compound	Mechanism of action	Validation	Reference
Benztropine	M1/M3 muscarinic acetylcholine receptor antagonism	Both in vitro and in vivo	[69]
Clemastine	M1 muscarinic acetylcholine receptor antagonism	Both in vitro and in vivo	[70]
Miconazole	MAPK/ERK1/2 signaling	Both in vitro and in vivo	[74]
Clobetasol	Glucocorticoid receptor signaling	Both in vitro and in vivo	[74]
Solifenacin	M3 muscarinic acetylcholine receptor antagonism	In vivo	[73]
(±)-U50488	κ-opioid receptor agonism	Both in vitro and in vivo	[75]
Bazedoxifene	Unknown	Both in vitro and in vivo	[76]
Danazol	p57kip2 protein modulation	Both in vitro and in vivo	[79]
Parbendazole	p57kip2 protein modulation	Both in vitro and in vivo	[79]
Ro1138452	Unknown	In vitro	[81]
SR2211	Unknown	In vitro	[81]
ESI1	Epigenetic rejuvenation	Both in vitro and in vivo	[84]
Leucovorin	Unknown	Both in vitro and in vivo	[82]
Dyclonine	Unknown	Both in vitro and in vivo	[82]
PIPE-307	M1 muscarinic acetylcholine receptor antagonism	Both in vitro and in vivo	[72]

More importantly, selective depletion of *Chrm1*, the gene encoding the M1 muscarinic acetylcholine receptor in OPCs, led to a remarkable increase in the expression of mature myelin markers in OPC cultures and accelerated remyelination following local demyelination in mice [71]. Based on these findings, muscarinic antagonism is increasingly being explored as a potential strategy for the development of remyelination therapies. A recent study has identified a new brain-penetrable small molecule antagonist of the M1 muscarinic acetylcholine receptor, named PIPE-307 [72]. PIPE-307 treatment significantly enhanced OPC differentiation and myelination in OPC cultures, mouse cortical slice cultures, and human cortical slice cultures. Furthermore, it significantly increased the number of remyelinated axons following in vivo demyelination. Other muscarinic acetylcholine receptor subtypes may also serve as targets for remyelination therapy. Systemic administration of solifenacin, an FDA-approved M3 muscarinic acetylcholine receptor antagonist, has been shown to accelerate myelination in mice and enhance the differentiation and myelination of transplanted human OPCs in the brains of hypomyelinated shiverer mice [73].

Other pro-myelinating compounds with distinct targets have also been reported. Through screening a library of bioactive small molecules on mouse pluripotent epiblast stem-cell-derived OPCs, two pro-myelinating agents, miconazole and clobetasol, were identified. Both drugs were found to effectively promote the generation of mature oligodendrocytes from mouse and human OPCs and enhance myelination in organotypic cerebellar slice cultures, early postnatal mouse pups, and focal demyelination mouse models [74]. Moreover, genome-wide RNA sequencing and phosphoproteomic analyses revealed that miconazole and clobetasol function in OPCs via the MAPK/ERK1/2 and glucocorticoid receptor-mediated signaling pathways, respectively [74]. Besides its pro-myelinating properties, clobetasol was also found to exert immunosuppressant effects, whereas miconazole was shown to act directly as a remyelination agent without affecting the immune system [74]. Mei et al. [75] conducted a screening of a G-protein-coupled receptor small-molecule library, leading to the identification of a cluster of κ-opioid receptor (KOR) agonists with pro-myelinating properties. Among this cluster, the small-molecule drug U-50,488 was shown to be the most effective in promoting OPC differentiation in vitro. U-50,488 treatment also significantly enhanced remyelination in vivo following the induction of focal demyelination. Intriguingly, conditional deletion of KOR in OLs largely abolished the pro-myelinating effects of U-50,488, indicating that its mechanism of action involves agonism of the κ-opioid receptor in OLs [75]. Bazedoxifene, a selective estrogen receptor modulator, has been found to exhibit pro-myelinating properties independent of its action on estrogen receptors [76]. In both in vitro and in vivo models, bazedoxifene was shown to enhance the differentiation of OPCs into functional oligodendrocytes. Notably, these effects were observed in the absence of estrogen receptors, including estrogen alpha and beta receptors, indicating that its pro-myelinating action is not mediated by estrogen receptors. Instead, subsequent bioinformatics profiling identified emopamil-binding protein (EBP), a key enzyme in cholesterol biosynthesis, as a potential mediator of its effects [76]. However, as G protein-coupled estrogen receptor 1 (GPER), previously known as GPR30, also mediates rapid cellular signaling in response to estrogen [77, 78], it remains to be determined whether bazedoxifene can act on GPER to influence myelination.

Given that the p57kip2 protein has been identified as an intrinsic negative regulator of OPC differentiation and oligodendroglial maturation [79], Manousi et al. [80] conducted a phenotypic compound screening targeting the subcellular distribution of p57kip2 in primary rodent OPCs. This screening led to the discovery of two novel small molecules capable of promoting OPC differentiation: the steroid danazol and the anthelmintic parbendazole. Subsequent in vivo experiments demonstrated the efficacy of danazol and parbendazole in enhancing remyelination in a cuprizone-induced demyelination model [80].

Using a human stem cell-derived OPC-based high-throughput drug screening platform, two small molecules—Ro1138452, a selective prostacyclin receptor antagonist, and SR2211, a retinoic acid receptor-related orphan receptor gamma modulator—were identified as promoters of OPC differentiation and oligodendrocyte maturation [81]. Gene set enrichment analysis revealed an upregulation of genes associated with sterol and cholesterol biosynthesis pathways, which are critical for oligodendrocyte maturation and myelination, in the oligodendrocyte cluster following treatment [81]. These compounds appear to exert their pro-myelinating effects through non-canonical pathways, as transcriptomic analysis revealed very low expression levels of their canonical targets in the OLs [81]. Thus, the precise mechanisms through which these compounds promote myelination, remain to be elucidated, and their efficacy in animal models has yet to be determined. Through in vitro neural and OPC co-cultures, leucovorin and dyclonine, two FDA-approved medications, were shown to promote oligodendroglial fate acquisition, OPC differentiation, and oligodendrocyte maturation [82]. Treatment with these small molecules enhanced the pro-oligodendrogenic activity of neural progenitor cultures, as evidenced by an increased number of OPCs and mature oligodendrocytes. Furthermore, their pro-myelinating properties were validated in various animal models of demyelination, although the exact molecular mechanisms underlying these actions remain to be elucidated [82].

Notably, recent findings suggest that epigenetic silencing in both oligodendrocytes and Schwann cells can hinder myelination [83, 84]. These observations raise the possibility that epigenetic rejuvenation could serve as a promising target for promoting myelin regeneration. In this context, Liu et al. recently identified a small-molecule epigenetic silencing inhibitor—ESI1, which has been shown to lengthen myelin sheaths in human iPSC-derived organoids and promote remyelination in different animal models of demyelination [84]. Further analysis revealed that ESI1 leads to the formation of nuclear condensates of key lipid metabolism regulators SREBP1/2, which concentrate transcriptional co-activators to promote the expression of key regulators of lipid and cholesterol biosynthesis, as well as myelinogenic regulators, in OLs [84].

Altogether, with an enhanced understanding of the complex intrinsic and extrinsic pathways involved in the development of OLs, it is anticipated that an increasing number of pro-myelinating compounds will be identified in the near future.

### Myelin dysfunction in aging and brain disorders

**Fig. 3:**
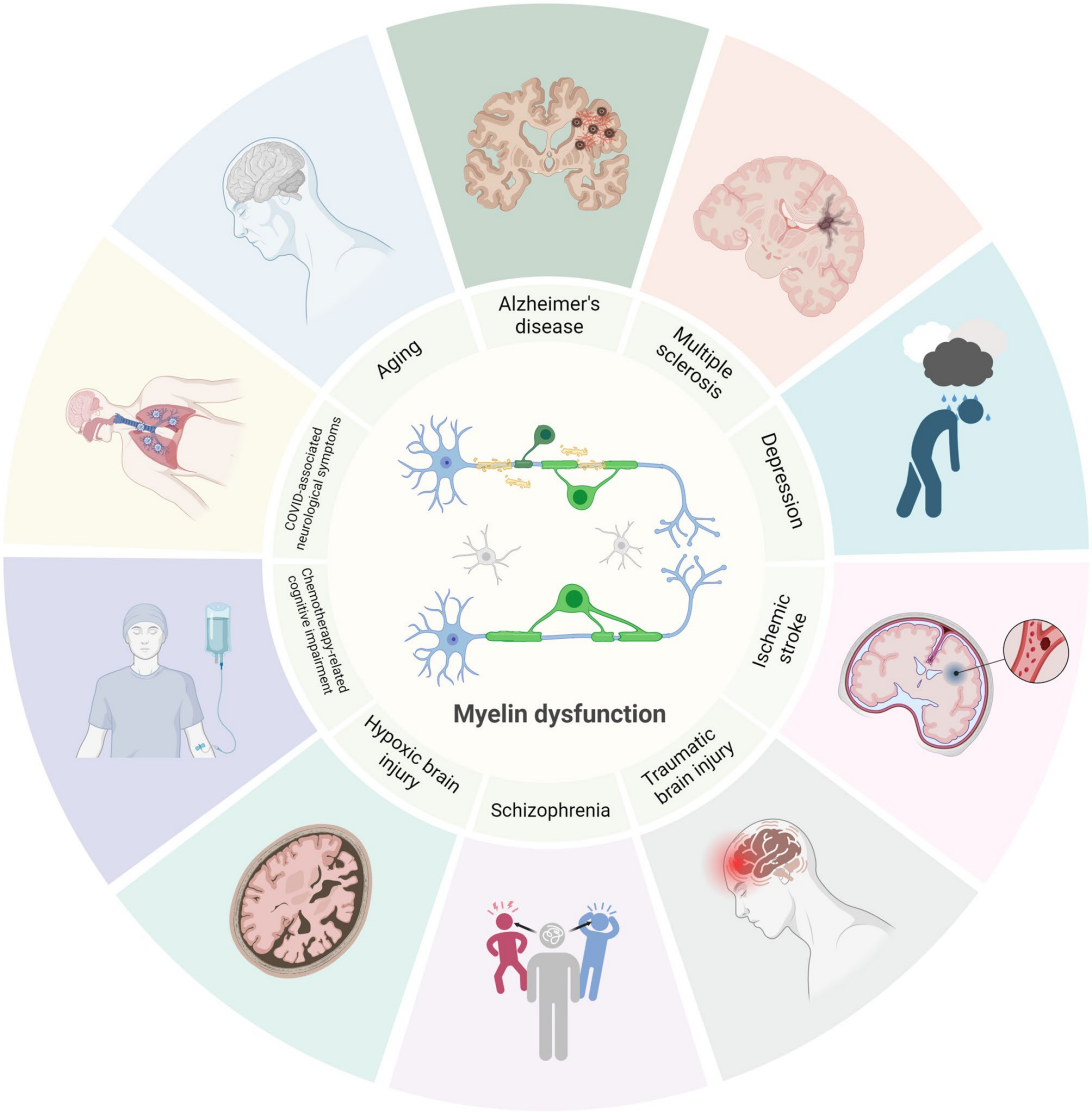
Myelin dysfunction in aging and brain disorders. The schematic illustrates myelin dysfunction in the aging brain and various brain disorders. These conditions often involve demyelination, hypomyelination caused by reduced new myelin formation, or a combination of both. Such myelin dysfunction may underlie the neurological symptoms commonly observed in these medical conditions

Accumulating evidence highlights myelin dysfunction in aging and various brain disorders (Fig. 3), typically involving demyelination, deficits in new myelin formation, or both. In the following sections, we review myelin dysfunction in these conditions and discuss the potential benefits of remyelination therapies.

### Myelin dysfunction and remyelination therapies in aging

With population aging, age-associated cognitive decline poses a significant challenge to the elderly. Even healthy individuals may experience subtle cognitive changes as they age [85, 86]. Indeed, several neuroimaging studies have revealed a decline in myelination across various brain regions with age in cognitively unimpaired individuals spanning a wide age range [87, 88]. Lower myelin levels in the prefrontal white matter, corpus callosum, and posterior limb of the internal capsule have been linked to reduced memory performance, even after adjusting for age, sex, and education [89]. Using high-resolution intravital label-free and fluorescence optical imaging, a study revealed lifelong oligodendrocyte and myelin dynamics in the mouse cortex [90]. Following peak myelination, gradual oligodendrocyte death, myelin degeneration, pronounced internode loss, and the accumulation of myelin debris were observed during advanced aging [90]. These findings are further supported by studies in non-human primates, which demonstrate reduced myelin levels across various brain regions with aging [91–93]. Considering the essential role of myelin in memory and cognitive function, this observation raises the intriguing possibility that age-related myelin dysfunction may contribute to cognitive decline associated with aging. In this context, Wang et al. [94] provided novel insights into age-related alterations in myelin dynamics. By using inducible Cre/loxP system-based transgenic mice, it was found that newly formed myelin sheaths in the cortex of aged mice were significantly reduced compared to those in young mice. Myelin sheaths generated before 3 months of age were also diminished in 14-month-old mice when compared to 4-month-old mice, indicating that aging not only diminishes myelination but also contributes to myelin degeneration. Table 2 summarizes studies that explore myelin breakdown associated with aging.

**Table 2:** Studies of Myelin dysfunction in aging and alzheimer’s disease

Conditions	Species	Main findings	Reference
Aging	Mouse	After peak myelination, gradual oligodendrocyte death, myelin degeneration, pronounced internode loss, and myelin debris accumulation occur during advanced aging in the cortex of mice.	[90]
		Aged mice show diminished myelin renewal and extensive myelin degeneration in the cortex compared to young mice.	[94]
		Aged mice exhibit reduced transcriptional activation and increased epigenetic silencing in oligodendrocytes across various brain regions. Epigenetic rejuvenation enhances myelination and alleviates age-associated cognitive deficits.	[84]
	Monkey	Aged rhesus monkeys show a 20% reduction in the density of myelinated nerve fibers in the cingulate bundle and genu of the corpus callosum compared to young controls.	[91]
		Aged common marmosets exhibit reduced myelin thickness, density, and myelin fraction in the corpus callosum compared to young adult marmosets.	[92]
		Aged rhesus monkeys exhibit reduced levels of myelin-associated proteins in the cingulum bundle and corpus callosum, which is correlated with poorer cognitive performance.	[93]
	Human	Brainstem myelin content declines with normal aging in cognitively unimpaired individuals aged 21 to 94.	[87]
		White matter myelin content declines with normal aging in cognitively unimpaired individuals aged 20 to 99.	[88]
		Lower myelin levels in the prefrontal white matter, genu of the corpus callosum, and posterior limb of the internal capsule are associated with reduced memory performance in healthy individuals aged 20 to 79.	[89]
AD	Mouse	R1.40 transgenic AD mice exhibit myelin loss in the neocortex, which occurs prior to AD-associated neurodegenerative changes.	[9]
		APP/PS1 transgenic AD mice exhibit defects in myelin integrity and reduced myelin content in the cortex at 6 months of age, which normalize by 9 months, accompanied by increased proliferation and differentiation of OPCs.	[106]
		APP/PS1 transgenic AD mice exhibit myelin loss in the corpus callosum as early as 3 to 6 months of age, accompanied by decreased levels of myelin-associated proteins in the temporal lobe.	[104]
		5xFAD transgenic AD mice show myelin breakdown in the corpus callosum at 12 months of age.	[105]
		Despite widespread myelin degradation in the hippocampus and cortex, 13–14-month-old APP/PS1 mice exhibit increased new myelin formation. Enhancing myelination, either genetically or pharmacologically, mitigates cognitive deficits associated with AD without affecting Aβ pathology.	[108]
		The APOE4 allele, the primary genetic risk factor for AD, impairs myelination by disrupting cholesterol metabolism. Pharmacological enhancement of cholesterol transport boosts axonal myelination and improves learning and memory in APOE4 mice.	[119]
		Young male P301S tau mice exhibit pronounced myelin loss in the hippocampal dentate gyrus, an effect mediated by X-linked TLR7 signaling.	[122]
	Human	AD patients exhibit reduced myelin integrity in the cerebral white matter.	[7]
		AD patients exhibit reduced myelin levels in the left temporal/parietal regions and bilateral periventricular normal-appearing white matter.	[8]
		Postmortem examinations of brains from AD patients reveal a loss of oligodendrocytes and key myelin proteins in the prefrontal cortex.	[9]
		Postmortem examinations of brains from AD patients reveal widespread myelin loss and degradation in the white matter.	[107]

AD, Alzheimer’s disease; OPCs, Oligodendrocyte precursor cells; TLR7, Toll-like receptor 7

Further studies demonstrated that impairing myelination through the deletion of Olig2, a critical transcription factor for the development of OLs, in OPCs, results in spatial memory deficits in young mice [94]. Conversely, enhancing myelination by deleting muscarinic acetylcholine receptor 1, a negative regulator of OPC differentiation, in OPCs, rescued memory deficits in aged mice. Intriguingly, a 4-month treatment with the pro-myelinating agent clemastine fumarate, initiated at 12 months of age, reversed aging-associated myelination deficits and rescued spatial memory decline during aging [94]. These findings suggest that remyelination therapy could be a promising strategy to counter cognitive decline during aging.

The mechanisms driving aging-associated myelin dysfunction may be multifaceted. Aging-associated alterations in the brain microenvironment likely play a key role. While OPCs isolated from aged rats exhibited low proliferation rates, these cells showed increased proliferation and differentiation—comparable to young OPCs—when transplanted into neonatal rat brains [95]. Notably, it was found that the brain extracellular matrix stiffens with aging, correlating with OPC dysfunction. However, aged OPCs cultured on synthetic scaffolds mimicking the stiffness of young brain extracellular matrix displayed remarkably enhanced proliferation and differentiation [95]. In line with this, the infusion of cerebrospinal fluid (CSF) from young mice into the aged brain enhanced hippocampal myelination [96]. In vitro experiments further showed that aged OPCs treated with young CSF exhibited increased proliferation and differentiation [96]. Beyond microenvironmental changes, OPCs undergo significant transcriptomic alterations during aging. Transcriptional profiling of mouse OPCs across the lifespan has identified signaling pathways that may regulate OPC proliferation [97]. Hif1a gene expression, which encodes HIF-1α, was found to increase with aging in mouse cortical OPCs. Intriguingly, pharmacological inhibition of HIF-1α restored the differentiation capacity of OPCs from aged mice, suggesting that age-related HIF-1α activation may contribute to impaired OPC differentiation [97]. A recent study provided important mechanistic insights into how aging affects myelination. Compared to young adult mice, oligodendrocytes in aged mice exhibited higher levels of HDAC3, an epigenetic repressor, while the levels of H3K27ac—an epigenetic mark associated with active enhancers and transcriptional activation—were significantly reduced in the corpus callosum and cortex [84]. Notably, a one-month daily treatment with the small-molecule epigenetic silencing inhibitor ESI1 in aged mice significantly increased the myelinated area in the cortex and hippocampus, accompanied by improved spatial memory compared to vehicle-treated aged animals [84]. These findings suggest that epigenetic silencing in oligodendrocytes may be an important contributor to the failure of myelination during aging. Epigenetic rejuvenation could, therefore, represent a promising therapeutic approach to promote remyelination in the aging brain.

Overall, aging-associated myelin dysfunction arises from complex and multifaceted mechanisms that may overlap with the pathophysiology of other demyelinating conditions. A deeper understanding of the mechanisms underlying aging-related myelin dysfunction may therefore offer new therapeutic opportunities for a range of demyelinating conditions, particularly those in which aging is a major risk factor.

### Myelin dysfunction and remyelination therapies in alzheimer’s disease

As one of the most prevalent age-related neurodegenerative disorders, AD is characterized by amyloid-beta (Aβ) plaque deposits, tau neurofibrillary tangle formation, and progressive cognitive decline [98, 99]. Historically, therapeutic strategies for this disease have been focused on Aβ or tau-targeting immunotherapies [100]. Nevertheless, to date, only a few such strategies have successfully resulted in disease-modifying therapies for AD, including Lecanemab [101] and Donanemab [102], both recently approved by the FDA. The disappointing outcomes of most immunotherapies in clinical trials have prompted researchers to reevaluate the etiology of this disease and explore alternative disease-modifying approaches [100, 103].

Emerging evidence suggests that myelin dysfunction may be an early pathological event and a significant contributor—independent of Aβ and tau—to the pathogenesis of AD (Table 2). Loss of myelin has been widely reported in the brains of both experimental AD models [9, 104–106] and AD patients [7–9, 107]. Neuroimaging and postmortem studies have revealed widespread myelin loss and white matter dysfunction across various brain regions in AD patients [7–9]. In a transgenic mouse model of AD, cortical myelin loss was found to precede neuronal degeneration and the onset of cognitive impairment [9]. Interestingly, in transgenic AD mouse models, despite the degradation of myelin, the AD brain, unlike the naturally aging brain, exhibits increased new myelin formation [108]. However, increased myelin formation observed in AD fails to result in the stable accumulation of new myelin sheaths [108]. Although it remains unknown why the AD brain exhibits enhanced myelin renewal, this alteration may represent an endogenous response to counter Aβ toxicity, as myelin defects have been shown to exacerbate Aβ deposition by influencing APP processing [109]. More importantly, enhancing new myelin formation genetically rescued memory deficits in AD mice, accompanied by increased hippocampal neuronal activity in response to memory tasks, despite the presence of microgliosis and Aβ pathology [108]. These findings strongly suggest that enhancing myelin may represent an additional therapeutic strategy for AD, independent of Aβ.

Several mechanisms have been proposed to explain myelin dysfunction associated with AD pathogenesis, including DNA damage response, Aβ toxicity, iron/copper dyshomeostasis, and cholesterol dysregulation, which have been thoroughly discussed in a previous review [110]. DNA damage in OLs has been identified as a pronounced event in the progression of AD, with substantial DNA damage and OL degeneration reported in postmortem AD brains [9]. Furthermore, experimentally inducing DNA damage in OL cultures markedly reduced the number of mature OLs, highlighting its critical role in myelin dysfunction [9]. As a major contributor to AD pathogenesis, Aβ toxicity may also directly contribute to myelin dysfunction. Although Aβ oligomer treatment has a minimal effect on OPC survival, it significantly reduces the survival of mature OLs [111]. A more recent study further elucidated how Aβ can trigger myelin dysfunction. In various mouse models of amyloidosis, a significantly elevated number of CD8^+^ T cells was observed in both white and gray matter [112]. Notably, depletion of these cells, either through antibody treatment or genetic manipulation, rescued the observed myelin abnormalities [112]. In addition, metal ions such as copper and iron, which are essential for proper myelin growth, maintenance, and function, have been reported to be dyshomeostatic in the brains of individuals with AD [113, 114]. Given the evidence that dyshomeostasis of brain copper and iron can directly lead to myelin dysfunction [115, 116], it is tempting to speculate that the dyshomeostasis of these metal ions may serve as a critical driver of myelin abnormalities in AD.

There is also strong evidence that AD brains exhibit cholesterol dysregulation [117]. Given the critical role of cholesterol metabolism in myelination [118], abnormalities in brain cholesterol metabolism may also be an active player in AD-associated myelin dysfunction. A recent study elegantly demonstrated how the APOE4 allele, the primary genetic risk factor for AD, affects the physiological process of myelination by disrupting cholesterol metabolism [119]. Analysis of postmortem human brains revealed that APOE4 carriers exhibit thinner myelin sheaths and fewer myelinated axons in the prefrontal cortex compared to age-matched non-carriers, along with increased intracellular cholesterol accumulation. Single-cell transcriptomic profiling further showed that the APOE4 allele significantly alters signaling pathways involved in cholesterol transport and cellular cholesterol homeostasis [119]. In cultured oligodendrocytes, APOE4 expression led to intracellular cholesterol accumulation and compromised myelination—effects that were rescued by enhancing cholesterol transport [119]. Enhancing cholesterol transport through pharmacological means also promoted axonal myelination and improved learning and memory in APOE4 mice [119].

In addition, senescence in OPCs may contribute to the dysfunctional myelin physiology observed in AD. Brains from AD patients and transgenic mouse models of AD exhibit a senescence-like phenotype in OPCs located near Aβ plaques [120]. The exposure of OPC cultures to Aβ could directly induce senescence in OPCs [120]. This senescent phenotype may impair the differentiation capacity of OPCs, leading to compromised myelin renewal at certain stages of the disease. The deterioration of the local microenvironment, driven by neuroinflammation, oxidative stress, and the accumulation of toxic proteins, may also compromise the regenerative capacity of OPCs, as reviewed elsewhere [121]. A more recent study demonstrated that tau pathology can induce demyelination in a sex-dependent manner. In 3- to 4-month-old male tau transgenic (P301S tau) mice, a pronounced loss of myelin was observed in the hippocampal dentate gyrus, whereas female mice showed no such effect [122]. Tau-induced demyelination appears to involve X-linked toll-like receptor 7 (TLR7) signaling, as TLR7 inhibition protected male mice from tau pathology-associated demyelination [122].

New evidence indicates that several pro-myelinating agents exhibit beneficial effects in mitigating learning and memory deficits in rodent models of AD. For instance, clemastine fumarate, administered daily to 8-month-old APP/PS1 mice for 3 months, significantly enhanced new myelin formation in the brain [108]. This was accompanied by improved performance in memory-related tasks and increased neuronal activity following task completion, without altering Aβ deposition or clearance [108]. In line with these findings, the therapeutic benefits of the pro-myelinating agent miconazole in AD have been reported. In a mouse model of AD, miconazole treatment enhanced oligodendrocyte maturation and increased myelin sheath thickness in the mPFC [123]. These changes were accompanied by improved performance in cooperative deficit tasks, an early manifestation of AD. Notably, Aβ plaque deposition, reactive gliosis, and inflammatory factor levels remained unchanged. Similarly, miconazole treatment reversed the Aβ^1–42^-induced reductions in oligodendrocyte maturation in OL cultures [123].

It should be noted that AD is a complex and multifactorial disorder, and its heterogeneity may complicate the interpretation of these findings. Indeed, 99% of clinical AD cases are sporadic, with only a small fraction being familial AD [124]. Increasing evidence highlights distinct pathological alterations between familial and sporadic AD [125–127]. Nevertheless, how these differences potentially affect oligodendrocyte and myelin pathophysiology remains largely unknown. Bridging this knowledge gap, therefore, is crucial for developing effective remyelination therapies for AD patients.

### Myelin dysfunction and remyelination therapies in multiple sclerosis

MS is an inflammatory, autoimmune demyelinating disease characterized by an aberrant immune response that leads to the loss of the myelin sheath and axonal damage in both the spinal cord and brain [128, 129]. As a result, immunomodulatory treatments and remyelination therapies have been the primary focus of MS management over the past decades [130, 131]. Despite variability in disease progression, subtypes, and symptoms, patients with MS typically experience clinical manifestations such as fatigue, muscle stiffness and tremors, coordination and balance issues, impaired visual function, and cognitive impairments [129, 132, 133]. Multiple lines of evidence from brain imaging [10, 134, 135], postmortem studies [11, 136–138], and preclinical animal research [12, 84, 139, 140] have implicated widespread brain demyelination in MS (Table 3). A study compared brain myelin pathology between patients with different types of MS [135]. It was found that both relapsing-remitting and progressive MS patients exhibited reduced myelin content and axonal damage in the normal-appearing white matter and cortical lesions compared to healthy controls. Notably, patients with progressive MS showed more extensive loss of myelin and axonal integrity in the normal-appearing cortex than those with relapsing-remitting MS [135]. Another study examined longitudinal changes in brain myelin in patients with relapsing-remitting MS using neuroimaging [134]. Over five years, the mean myelin water fraction in normal-appearing white matter decreased by 8%, further supporting the notion that myelin integrity changes and myelin loss can occur diffusely and progressively in MS brains [134]. Experimental autoimmune encephalomyelitis (EAE) is the most commonly used animal model for MS. It is induced either through subcutaneous immunization with myelin peptides in an adjuvant, combined with the intravenous inoculation of *Bordetella pertussis* toxin or via the adoptive transfer of encephalitogenic T cells [141, 142]. This model replicates both the pathological and clinical features of MS. In animal models of EAE, widespread demyelination has been observed in the spinal cord as well as various brain regions and optic nerve [84, 139, 140].

**Table 3:** Studies of Myelin dysfunction in multiple sclerosis

Species	Main findings	Reference
Mouse	Mice subjected to EAE exhibit widespread demyelination lesions in various brain regions, including the cortex, hippocampus, internal capsule, cerebellum, and optic nerve, along with axonal damage.	[12]
	Mice subjected to EAE show demyelination and axonal damage during the early, middle, and late phases of the disease.	[139]
	Mice subjected to EAE exhibit widespread demyelination in the cortex and callosal white matter, accompanied by significant axonal degeneration.	[140]
	Epigenetic rejuvenation, achieved through either pharmacological approaches or genetic manipulation, enhances myelination in the optic nerve and promotes functional recovery in mice subjected to EAE.	[84]
Human	Postmortem brains from patients with different types of MS show myelin loss in the white matter or the normal-appearing white matter.	[11]
	Postmortem analysis of brains from patients with secondary progressive MS reveals significant myelin loss in white matter lesions.	[136]
	Postmortem brains from patients with early MS show active remyelination in cerebral lesions, compared to those from patients with chronic MS.	[137]
	Postmortem brains from patients with secondary progressive MS exhibit greater overall demyelination and higher brain loads of active demyelination compared to those with primary progressive MS.	[138]
	Patients with primary progressive MS exhibit lower myelin levels throughout the brain. Reduced myelin levels in the corpus callosum and frontal, temporal, parietal, and occipital white matter are correlated with increased clinical disability.	[10]
	Patients with relapsing-remitting MS exhibit reduced myelin levels in normal-appearing white matter.	[134]
	Patients with relapsing-remitting MS and progressive MS exhibit reduced myelin content and axonal damage in both normal-appearing white matter and cortical lesions. However, patients with progressive MS show more extensive loss of myelin and axonal integrity in the normal-appearing cortex compared to those with relapsing-remitting MS.	[135]
	Clemastine treatment reduces the latency of visual-evoked potentials—a measure reflecting the speed of neural conduction and indirectly indicating the health of myelin in the optic nerve—in patients with MS.	[148]

EAE, Experimental autoimmune encephalomyelitis; MS, Multiple sclerosis

Remyelination can occur at all stages of MS, however, endogenous remyelination within lesions is often transient and inadequate, which leads to overall axonal degeneration and clinical disability [143–145]. Despite the presence of OPCs in lesional regions, mature oligodendrocytes are significantly reduced or even absent in these areas [146, 147]. This deficiency is likely due to the inhibitory microenvironment within the lesions, which impairs OPC differentiation. Recent pioneering research has uncovered a crucial mechanism underlying the failure of remyelination in MS [84]. Postmortem brain tissue analysis from MS patients revealed that the overall number of OLs within lesions is comparable to that in adjacent, normal-appearing white matter. However, OLs in the lesions exhibited a state of epigenetic suppression without obvious apoptosis. In contrast, epigenetic rejuvenation, achieved through either pharmacological approaches or genetic manipulation, significantly enhanced myelination in the optic nerve and promoted functional recovery in a mouse model of EAE [84]. This observation further supports the idea that enhancing OPC differentiation could serve as a promising therapeutic strategy for MS.

To date, the identification of pro-myelinating compounds has primarily focused on their therapeutic potential for MS. Most of these compounds, including benztropine [69], miconazole [74], clobetasol [74], clemastine fumarate [71], ESI1 [84], and PIPE-307 [72], have been tested in experimental models of MS, yielding encouraging results. It is important to note that most of these studies utilize the EAE model, which induces demyelination in both the spinal cord and certain brain regions. However, these studies primarily focus on assessing myelination within spinal cord lesions. The efficacy of these pro-myelinating compounds in enhancing brain myelination and their potential to alleviate other functional deficits, such as cognitive impairments and visual deficits linked to brain demyelination, remains to be determined. Clinically, a double-blind, randomized, placebo-controlled, crossover trial has demonstrated the safety and efficacy of clemastine fumarate in patients with MS [148]. In this trial, 50 patients with relapsing MS, mild neurological disability, and chronic optic neuropathy underwent a 90-day treatment with different clemastine fumarate fumarate regimens. The outcome measures revealed that clemastine fumarate significantly reduced visual-evoked potential latency, a measure reflecting the speed of neural conduction and indirectly indicating the health of myelin in the optic nerve, in patients with MS, with only mild adverse events reported [148]. These landmark findings further support the potential of remyelination therapy in alleviating MS-associated disabilities.

### Myelin dysfunction and remyelination therapies in depression

Depression is a highly prevalent and disabling disorder that affects over 300 million individuals globally [149]. The World Health Organization ranks it as the primary contributor to disease-related disability worldwide [150]. Furthermore, the prevalence of depression is expected to rise, partly driven by the prolonged effects of the pandemic [151].

Myelin dysfunction and white matter abnormalities have been linked to the pathogenesis of depression (Table 4). Postmortem brains from patients with major depressive disorder showed reduced myelin levels in the dorsolateral prefrontal cortex [152]. Compared to healthy individuals, patients with major depressive disorder exhibit lower levels of myelin in most cortical regions [153–155]. Additionally, reduced myelin levels in certain regions have been inversely associated with the number of depressive episodes [153, 156]. Of note, a causal relationship between depression and myelin dysfunction cannot be established from these observations, as it remains unclear whether the decreased brain myelin levels in patients with depression are a consequence of the disease or if lower myelin levels increase susceptibility to depression. Experimental animal models provide further insight into this association. Reduced myelin levels in the mPFC have been reported in mice subjected to chronic social defeat, accompanied by a significant reduction in myelin-associated gene expression quantified by microarray analysis [157]. Similarly, a reduction in myelinated fibers has been observed in the white matter and hippocampus in experimental models of depression induced by unpredictable stress and lipopolysaccharide [13, 14, 158]. Of note, abnormalities in OLs may directly contribute to depressive-like behaviors in experimental animals. The ablation of Ninj2, a membrane adhesion molecule, in oligodendrocytes led to a reduced myelin area in the prefrontal cortex and hippocampus, as well as a decreased number of myelinated axons in the corpus callosum [159]. These structural changes were accompanied by depressive-like behaviors in mice [159].

**Table 4:** Studies of Myelin dysfunction in depression and schizophrenia

Conditions	Species	Main findings	Reference
Depression	Mouse	Mice subjected to chronic social defeat show reduced expression of myelin-associated genes in the mPFC.	[157]
		Mice subjected to CUS exhibit reduced expression of myelin-associated genes in the white matter tracts.	[13]
		Mice subjected to CUS exhibit reduced myelin sheath volume, axonal integrity, and the length of myelinated nerve fibers in the hippocampus.	[14]
		Mice subjected to CUS exhibit increased expression of EphA4 in the hippocampus, and its knockdown prevents CUS-induced demyelination, synaptic deficits, and depressive-like behaviors. Pharmacological enhancement of myelination also rescues myelin loss and alleviates depressive-like behaviors associated with CUS.	[158]
	Human	Postmortem brains from patients with major depressive disorder show reduced myelin levels in the dorsolateral prefrontal cortex.	[152]
		Patients with major depressive disorder exhibit lower levels of myelin both at the whole-brain level and in the nucleus accumbens compared to healthy controls. Additionally, individuals with a greater number of depressive episodes show reduced myelin in the lateral prefrontal cortex.	[153]
		Patients with major depressive disorder show lower myelin content in the many cortical regions compared to healthy controls.	[156]
		Patients with major depressive disorder exhibit lower average sq-ratio myelin-related values in the white matter and subcortical regions compared to healthy controls.	[154]
		Patients with major depressive disorder have reduced myelin density in the IFOF and UF compared to healthy controls.	[155]
Schizophrenia	Rat	Schizophrenia-like rats exhibit decreased expression of myelin-related genes, a reduced number of mature oligodendrocytes, and fewer myelinated parvalbumin inhibitory axons in the medial prefrontal cortex, accompanied by cognitive deficits. Restoring parvalbumin interneuron hypomyelination in the medial prefrontal cortex rescues cognitive deficits.	[173]
	Mouse	Schizophrenia-like mice display reduced myelin protein expression in the cerebellum.	[171]
		Schizophrenia-like mice exhibit splitting of myelin sheath lamellae, segmental demyelination, and a reduced total length of myelinated fibers in the corpus callosum.	[172]
	Human	Patients with schizophrenia exhibit significantly reduced overall white matter myelin levels, particularly in the left genu of the corpus callosum. Postmortem analyses of the brains of schizophrenia patients reveal decreased levels of myelin-associated proteins in the anterior frontal cortex.	[170]
		Postmortem brains from patients with schizophrenia show reduced expression of key oligodendrocyte- and myelin-related genes in the prefrontal cortex.	[22]
		Postmortem brains from patients with chronic schizophrenia exhibit downregulated expression levels of myelin-associated genes in the dorsolateral prefrontal cortex.	[21]
		Patients with schizophrenia exhibit reduced myelin content and intra-axonal abnormalities in white matter compared to healthy controls.	[23]

EphA4, Ephrin A4 receptor; CUS: Chronic unpredictable stress; mPFC, medial prefrontal cortex; IFOF, Fronto-occipital fasciculus; UF, Uncinate fasciculus

A recent study provides valuable insights into the biological mechanisms underlying depression-associated myelin dysfunction. In animals exposed to chronic unpredictable stress, the expression of the ephrin A4 receptor (EphA4), a suppressor of neurotransmission and synaptic plasticity, was significantly elevated in excitatory neurons within the hippocampus [158]. Notably, knockdown of brain EphA4, either ubiquitously or specifically in excitatory neurons, effectively prevented demyelination, synaptic deficits, and depressive-like behaviors induced by chronic stress exposure. These findings highlight EphA4 as a critical mediator linking depression to myelin dysfunction [158]. Supporting the idea that enhancing myelination can reverse behavioral changes in mouse models of depression, Li et al. demonstrated that clemastine fumarate treatment significantly rescued myelin loss in the hippocampus and alleviated depressive behaviors in these models [158]. These observations establish a causal relationship between myelin dysfunction and depression and suggest that restoring myelination may represent a promising therapeutic strategy for this mental disorder. Of interest, the antidepressant ketamine, including its enantiomers (R)-ketamine and (S)-ketamine, may exert long-lasting antidepressant effects by promoting myelination. (R, S)-ketamine treatment significantly enhanced myelination in the prefrontal cortex and hippocampus of mice subjected to chronic social defeat stress. However, the knockdown of Mobp, a structural protein critical for myelin stability, abolished the long-lasting antidepressant effects of (R, S)-ketamine [160]. Additionally, (R)-ketamine was found to promote myelination more effectively than (S)-ketamine, which may explain the stronger and longer-lasting antidepressant effects of (R)-ketamine compared to (S)-ketamine [160].

In summary, targeting myelin appears to be a promising therapeutic approach for managing depression. However, from a clinical perspective, depressive episodes often require immediate treatment, it remains unclear how quickly patients would benefit from remyelination therapies and how long those benefits would last, which warrants further investigation.

### Myelin dysfunction in schizophrenia

Schizophrenia is a severe mental illness characterized by delusions, hallucinations, formal thought disorder, and impaired executive functioning [161]. The onset of this disorder typically occurs in early adulthood. However, most individuals with schizophrenia show prodromal symptoms, including progressive changes in thinking, mood, and social functioning, preceding the first episode of psychosis [161, 162]. Over the past 30 years, the global prevalence, incidence, and disability-adjusted life years of schizophrenia have increased by 65%, 37%, and 65%, respectively, posing a significant burden on individuals and society [163, 164]. The pathogenesis of schizophrenia involves early-life environmental and genetic risk factors, although its exact etiology remains an enigma [165, 166]. Current therapies primarily target the blockade of dopamine D2 receptors. However, these pharmacological treatments often exhibit limited efficacy, and poor tolerability, and are associated with adverse effects [165, 167]. In light of these challenges, a better understanding of the pathological alterations underlying schizophrenia is crucial for identifying novel therapeutic targets.

Cognitive impairment is a core clinical feature of schizophrenia, thought to arise from abnormalities in brain connectivity, and contributes significantly to the functional impairments associated with the disorder [168, 169]. Indeed, multiple lines of evidence have implicated OL and myelin dysfunction in schizophrenia (Table 4). Postmortem studies have revealed that several key myelin-associated genes are transcriptionally downregulated in the prefrontal cortex of patients with chronic schizophrenia [21, 22]. Additionally, MAG and CNPase, two critical myelin-related proteins, were found to be reduced in the anterior frontal cortex of schizophrenia brains [170]. Neuroimaging studies further demonstrated reduced myelin content in specific brain regions of individuals with schizophrenia [23, 170]. Compared to age-matched healthy controls, individuals with schizophrenia exhibit significantly lower overall brain myelin levels, particularly in the frontal lobe white matter and the genu of the corpus callosum, where the myelin water fraction is reduced by 20% and 10%, respectively [170]. Similarly, reduced myelin levels and decreased expression of key myelin proteins have been observed across various brain regions in schizophrenia-like rodent models [171–173]. Decreased expression of myelin-related genes, a reduced number of mature oligodendrocytes, and fewer myelinated parvalbumin inhibitory axons have been observed in the mPFC in a rat model of schizophrenia, accompanied by mPFC-dependent cognitive deficits [173]. Environmental enrichment restored parvalbumin interneuron hypomyelination in the mPFC and rescued cognitive deficits in this schizophrenia-like rat model [173]. However, since environmental enrichment influences multiple cellular processes beyond myelination, it remains unclear whether the cognitive improvements resulted specifically from enhanced myelination or other mechanisms. Further studies using targeted genetic or pharmacological approaches to promote myelination in schizophrenia-like animal models are warranted to establish causality between hypomyelination and cognitive impairment in schizophrenia.

Of note, the myelin dysfunction observed in schizophrenia brains may originate from abnormalities in OPCs. OPCs derived from schizophrenia patients exhibit a diminished ability to differentiate into oligodendrocytes compared to those from healthy individuals [174]. Additionally, OPCs in schizophrenia patients display a distinct morphology characterized by excessive branching, which has recently been identified as a unique pathological phenotype [175, 176]. Introduction of the DISC1-Δ3 gene, a splicing variant of the schizophrenia-associated DISC1 gene, into OPCs induced this pathological phenotype in mice, along with abnormal synaptic formation, altered neuronal activity, and schizophrenic-like behaviors, without affecting myelination [175]. Thus, these findings raise the possibility that normalizing the pathological phenotype of OPCs and enhancing myelination could represent novel therapeutic strategies for schizophrenia, although direct evidence is currently unavailable.

### Myelin dysfunction and remyelination therapies in ischemic stroke

Stroke, a life-threatening condition, occurs when blood flow to the brain is disrupted—most commonly due to the blockage of a blood vessel caused by a clot or atherosclerosis, or less frequently, by a hemorrhage resulting from damage to blood vessels [177, 178]. It is also a leading cause of long-term disability in adults [178]. Overall, more than 70% of stroke cases are ischemic, with first-line management strategies including intravenous thrombolysis, endovascular thrombectomy, or a combination of both [178]. However, many ischemic stroke survivors experience chronic and permanent neurological deficits, such as motor dysfunction and cognitive impairment [179, 180], while effective treatments for these functional deficits remain unavailable. Thus, a deeper understanding of the mechanisms underlying secondary injury following stroke, as well as the development of targeted therapies, is urgently needed.

Evidence from experimental models of ischemic stroke and stroke survivors suggests the potential involvement of myelin dysfunction in the prolonged motor and cognitive deficits associated with ischemic stroke (Table 5). Extensive demyelination has been observed in the ischemic hemisphere across mouse, rat, and monkey models of ischemic stroke, occurring within days of ischemic injury and persisting for months [15, 16]. Additionally, in a mouse model of focal cortical ischemia, demyelination was detected in non-ischemic regions of the corpus callosum [181]. These findings highlight the susceptibility of myelin to ischemia and suggest that even localized ischemia may impact myelin integrity in distal brain regions. Studies in individuals with chronic stroke have also revealed reduced myelin contents in perilesional white matter [182, 183]. Since OLs are highly susceptible to oxygen and nutrient deprivation—conditions that can occur during ischemia due to interrupted cerebral blood flow—this vulnerability may explain the myelin loss observed in ischemic brains [54, 184].

**Table 5:** Studies of Myelin dysfunction in ischemic stroke and traumatic brain injury

Conditions	Species	Main findings	Reference
Ischemic stroke	Rat	Rats subjected to transient MCAO exhibit significant demyelination in the ischemic lesions at various time points following ischemic injury.	[15]
		Rats subjected to transient MCAO show dynamic cycles of demyelination, remyelination, and subsequent demyelination within ischemic lesions at different time points following ischemic injury.	[16]
	Monkey	Cynomolgus monkeys subjected to MCAO exhibit significant demyelination within the infarcts at 8 days post-MCAO, followed by progressive loss of myelin and axons one month after the MCAO.	[17]
	Mouse	LRP1 binds to lipocalin-2 in astrocytes, driving astrocytic phagocytosis of myelin and leading to demyelination following focal cortical ischemia.	[181]
		LDLR expression is reduced in the corpus callosum following chronic cerebral ischemia. Genetic enhancement of LDLR expression in oligodendrocytes mitigates myelin pathology associated with cerebral ischemia.	[185]
		Elevated EphA4 levels following cerebral ischemia promote OPC differentiation at the expense of OL maturation. Conditional knockdown of EphA4 in OPCs enhances remyelination after cerebral ischemia.	[186]
		The Kir4.1 channel is downregulated following cerebral ischemia. Selective knockdown of Kir4.1 in OPCs, but not in astrocytes, exacerbates demyelination in the infarct area. Conversely, augmenting Kir4.1 channel currents promotes remyelination and functional recovery after cerebral ischemia.	[187]
		Cerebral ischemia triggers myelin regeneration in lesions, albeit with limited efficiency. Inhibiting new myelin formation worsens motor and memory impairments associated with ischemic injury, whereas genetically or pharmacologically enhancing myelination alleviates long-term functional deficits resulting from cerebral ischemia.	[188]
	Human	Chronic stroke survivors exhibit reduced myelin content in the whole-cerebrum white matter, as well as in the ipsilesional and contralesional posterior limb of the internal capsule, compared to healthy controls.	[182]
		Chronic stroke survivors exhibit reduced myelin contents in perilesional white matter compared to healthy controls.	[183]
TBI	Rat	Rats subjected to TBI exhibit a significant reduction in myelinated axons across various brain regions in the traumatized hemisphere, which can be observed from a few days to 12 months after injury.	[198]
		Rats subjected to TBI show widespread myelin loss in the external capsule, fimbriae, and corpus callosum at 2, 7, and 21 days post-injury.	[200]
		Rats subjected to TBI exhibit myelin loss in the cortex and hippocampus. Pharmacological enhancement of myelination preserves neuronal axons and mitigates neuronal damage following TBI.	[210]
	Mouse	Mice subjected to TBI display widespread demyelination in peri-lesional regions.	[199]
		Mice subjected to single or repeated TBI exhibit reduced myelin protein expression in the hippocampus and structural alterations in the nodes of Ranvier within the corpus callosum.	[18]
		Mice subjected to TBI show a reduction in myelinated axons and an increase in degenerating axons within the corpus callosum.	[19]
	Human	Collegiate hockey players who sustained concussions during the past season of athletic competition show a temporary reduction in myelin levels across various brain regions.	[20]
		Individuals who experienced moderate to severe diffuse TBI show reduced white matter myelin levels at 3 months post-injury compared to healthy controls.	[201]
		Individuals with chronic mild TBI and persistent symptoms exhibit significantly reduced global white matter myelin levels compared to healthy controls.	[202]

MCAO, Middle cerebral artery occlusion; LRP1, Low-density lipoprotein receptor-related protein 1; LDLR, Low-density lipoprotein receptor; EphA4, Ephrin A4 receptor; OPCs, oligodendrocyte precursor cells; OL, oligodendrocyte lineage cell; TBI, Traumatic brain injury

Recent studies have revealed new mechanisms by which cerebral ischemia leads to myelin dysfunction. Although the overall cerebral cholesterol levels remained unchanged, the mRNA levels and protein expression of low-density lipoprotein receptor (LDLR)—a protein receptor critical for maintaining cholesterol homeostasis—were reduced in the corpus callosum following chronic cerebral ischemia [185]. Mice deficient in LDLR exhibited fewer myelinated axons and thinner myelin sheaths, whereas genetically enhancing LDLR expression in oligodendrocytes alleviated the myelin pathology associated with cerebral ischemia [185]. Intriguingly, although both are involved in the regulation of cholesterol homeostasis, low-density lipoprotein receptor-related protein 1 (LRP1) was found to bind to lipocalin-2 in astrocytes, driving astrocytic phagocytosis of myelin and leading to demyelination following focal cortical ischemia [181]. Ablation of either lipocalin-2 or LRP1 reduced astrocyte-mediated myelin engulfment and mitigated demyelination after cerebral ischemia [181]. The EphA4 receptor has also been implicated in remyelination failure following ischemic stroke. The mRNA levels and protein expression of EphA4 were significantly upregulated in the corpus callosum of ischemic brains [186]. Although this increase in EphA4 did not contribute to OL apoptosis, further studies revealed that elevated EphA4 promotes the proliferation of OPCs but inhibits their differentiation. Conversely, conditional knockdown of EphA4 in OPCs was found to enhance remyelination [186]. In addition, evidence suggests that altered expression of specific ion channels contributes to remyelination failure following ischemic stroke. The protein expression of the Kir4.1 channel, an inwardly rectifying K⁺ channel subtype, was remarkably reduced in the infarction area of both acute ischemic stroke patients and animals subjected to cerebral ischemia [187]. Selective knockdown of the Kir4.1 channel in OPCs, but not in astrocytes, aggravated demyelination in the infarct area, while the augmentation of Kir4.1 channel currents promoted remyelination and functional recovery following cerebral ischemia [187]. These findings suggest that multiple mechanisms contribute to myelin dysfunction after ischemic stroke.

Studies employing genetic manipulations further elucidated the contribution of myelination dysfunction to prolonged motor and cognitive deficits following ischemic stroke. While cerebral ischemia triggered myelin regeneration in lesions, the efficiency of new myelin formation after stroke remained relatively low [188]. Inhibiting new myelin formation exacerbated motor and memory impairments associated with ischemic injury, whereas genetically enhancing myelination alleviated long-term functional deficits, as demonstrated by improved performance in motor and memory-related tests, suggesting the importance of myelin regeneration [188]. Moreover, clemastine fumarate was shown to enhance myelination in lesions, improve motor and memory functions after ischemic stroke, and increase neuronal survival and activity in the infarct area [188]. These findings provide solid evidence linking myelin dysfunction to persistent functional deficits following ischemic stroke and highlight the therapeutic potential of pro-myelinating agents. Although demyelination has also been reported in hemorrhagic stroke/intracerebral hemorrhage [189, 190], it remains unclear whether myelin pathology in these conditions involves distinct mechanisms and whether remyelination therapy can improve functional outcomes. Further investigation is required to bridge this knowledge gap.

### Myelin dysfunction and remyelination therapies in TBI

Globally, TBI is one of the leading causes of injury-related deaths, with more than 50 million individuals sustaining TBI annually [191, 192]. This represents a significant public health concern. It was once regarded as an acute event, however, the long-term consequences of TBI have now been recognized. A substantial proportion of TBI survivors report persistent symptoms, including attention difficulties, memory problems, and social deficits [193, 194]. A 30-year prospective cohort study revealed that individuals with two or more incident TBIs experienced a more rapid average cognitive decline with aging [195]. Additionally, a history of TBI has been linked to an increased risk of cognitive impairment among older adults [196]. Although advances in emergency care have led to improved survival of TBI patients, no treatments currently exist to prevent the long-term disabilities associated with TBI [197].

Indeed, demyelination and axonal loss are prominent pathological features following TBI and may represent a promising therapeutic opportunity (Table 5). Extensive myelin loss has been observed in experimental animal models of both diffuse and penetrating TBI [18, 19, 198–200]. Additionally, reduced myelin has been reported in individuals with moderate to severe diffuse TBI through neuroimaging studies [20, 201, 202]. While the mechanisms triggering the demyelination process following TBI remain unclear, since myelination typically requires the presence of axons [203], the widespread axonal degeneration caused by mechanical forces in TBI may explain the potential failure of remyelination [204, 205]. Furthermore, long-term cerebral hypoperfusion, widely reported in both animal models and patients with TBI [206–208], could induce both oxygen and energy crisis in the brain, potentially disrupting the physiological processes of OLs. These various factors may act together following TBI, ultimately contributing to chronic myelin loss. A remarkable study demonstrated that mice subjected to TBI exhibited significantly increased circulating autoantibodies against myelin at various time points following injury, accompanied by an increase in CD8^+^ T cells in the ipsilateral brain [209]. Notably, the deletion of CD8^+^ T cells improved neurological outcomes after TBI [209]. Although direct evidence remains limited, these findings raise the possibility that aberrant immune responses may contribute to prolonged demyelination following TBI.

Using a rat model of diffuse TBI, we have reported that the pro-myelinating compound clemastine fumarate effectively promotes myelination, preserves neuronal axons, and mitigates neuronal damage following TBI, without affecting gliosis [210]. Rats received clemastine fumarate treatment also showed improved memory performance after TBI, highlighting remyelination as a promising therapeutic strategy for TBI [210]. Of note, TBI survivors often face a significantly higher risk of depression [211–213]. This increased incidence may be explained by demyelination, which is known to contribute to depressive-like symptoms. Additionally, TBI has been proposed as an environmental risk factor for AD [214, 215]. In light of the shared myelin pathology in TBI and AD, myelin dysfunction may serve as a key mediator linking TBI to the pathogenesis of AD. In this context, pro-myelinating therapies might hold promise for preventing post-TBI depression and neurodegeneration triggered by TBI, although further research is needed to validate this approach.

### Myelin dysfunction in other neurological conditions

In addition to aging, major neurological disorders, and psychiatric conditions, myelin dysfunction has also been reported in various other neurological conditions (Table 6). The potential role of myelin dysfunction in these conditions is explored in this chapter.

**Table 6:** Studies of Myelin dysfunction in other neurological conditions

Conditions	Species	Main findings	Reference
Hypoxic brain injury	Mouse	Mice exposed to hypoxia during the early postnatal period exhibit hypomyelination across various brain regions.	[25]
		Mice exposed to early postnatal hypoxia exhibit hypomyelination in the corpus callosum, cortex, and cerebellum, alongside motor and cognitive deficits.	[219]
		Mice exposed to hypoxia during the early postnatal period exhibit widespread hypomyelination, accompanied by synaptic loss and delayed motor deficits. Enhancing myelination, either through genetic manipulation or pharmacological approaches, effectively rescues functional deficits caused by hypoxic brain injury.	[24]
		Adult mice exposed to chronic hypoxia show impaired formation of new myelin, while the levels of existing myelin remain unchanged.	[221]
	Human	A patient with delayed post-hypoxic leukoencephalopathy exhibited improved white matter pathology and significant functional recovery after treatment with the pro-myelinating agent clemastine fumarate.	[25]
Chemotherapy-related cognitive impairment	Rat	Rats treated with chemotherapy agents showed persistent cognitive impairments, a decrease in OPC numbers, and reduced myelin protein expression in the corpus callosum.	[231]
		Rats exposed to chemotherapy show a reduction in oligodendrocyte lineage cells and myelin in white matter, accompanied by significant cognitive deficits.	[227]
	Mouse	Mice exposed to chemotherapy exhibit impaired adaptive myelination and cognitive deficits, which are driven by disruptions in BDNF/TrkB signaling.	[229]
		Mice exposed to chemotherapy exhibit persistent myelin and neurological deficits in the frontal cortex.	[226]
		Mice exposed to chemotherapy exhibit demyelination in the corpus callosum and cognitive impairment.	[230]
	Human	Postmortem brains from individuals who underwent chemotherapy show a reduced population of oligodendrocyte lineage cells in the frontal lobe compared to age-matched control subjects without chemotherapy exposure.	[226]
COVID-associated neurological symptoms	Mouse	Mice infected with SARS-CoV-2 show a reduced number of oligodendrocyte lineage cells and myelinated axons in the subcortical white matter.	[26]
	Human	A COVID-19 survivor exhibits sudden neurological impairment accompanied by the new onset of multiple, non-enhancing demyelinating lesions in both the brain and spine.	[239]

BDNF, Brain-derived neurotrophic factor; TrkB, Tropomyosin receptor kinase B

### Hypoxic brain injury

Hypoxic brain injury, which typically occurs in preterm infants, can result in white matter injury and delayed functional deficits, particularly motor impairments [216–219]. Although no axon loss was observed, widespread hypomyelination has been reported in a mouse model of neonatal hypoxic injury, accompanied by synaptic loss and delayed motor deficits [24]. Enhancing myelination, either through genetic manipulation or pharmacological approaches—such as clemastine fumarate or (±)-U50488, could effectively rescue the functional deficits caused by hypoxic brain injury [24]. Although the findings from preclinical studies are promising, motor abnormalities resulting from neonatal hypoxic injury usually have a certain latency before clinical manifestation [220]. Therefore, beyond preventative strategies, it is important to explore whether pro-myelinating compounds, when administered in a delayed time window after motor deficits have manifested, can still provide benefits, which would be of greater clinical significance.

Chronic hypoxia can lead to myelin damage and motor coordination deficits in adults. One study reported a case of delayed post-hypoxic leukoencephalopathy in which a patient developed white matter abnormalities following an ischemic event [25]. Treatment with the pro-myelinating agent clemastine fumarate, initiated 2 months after the hypoxic event and continued for 10 months, resulted in significant functional improvements [25]. MRI revealed pronounced improvement in white matter pathology, with nearly complete resolution of diffuse white matter hyperintensities in the patient [25]. A study conducted in adult mice further revealed that chronic hypoxia exposure does not damage existing myelin but significantly impairs the formation of new myelin [221]. Similarly, treatment with clemastine fumarate resulted in enhanced myelination and improved motor coordination in mice exposed to chronic hypoxia [221].

### Chemotherapy-related cognitive impairment

Cognitive impairment is commonly reported in cancer patients undergoing chemotherapy and can significantly impact their quality of life. White matter pathology, including the loss of myelinated axons, has been identified as an adverse consequence of chronic chemotherapy and may contribute to chemotherapy-related cognitive impairment [222, 223]. Neuroimaging studies have revealed reduced white matter integrity in patients receiving chemotherapy [224, 225]. Postmortem examinations of children and young adults who received chemotherapy also revealed a reduced number of OLs in the subcortical white matter compared to age-matched controls without a history of chemotherapy [226].

Evidence from preclinical studies further supports a causal relationship between chemotherapy and cognitive impairment [226–230]. Rats exposed to chemotherapy agents exhibited persistent cognitive deficits, along with a reduced number of OPCs and decreased myelin protein expression in the corpus callosum [231]. Using a clinically relevant regimen of the chemotherapy agent methotrexate and its antidote leucovorin, a study reported the long-term effects of chemotherapy in rats. The results showed that rats treated with methotrexate and leucovorin exhibited reduced OLs and myelin in white matter, alongside pronounced cognitive deficits [227]. Another study elegantly revealed the mechanisms underlying chemotherapy-induced cognitive impairment. It was first discovered that brain-derived neurotrophic factor (BDNF)/TrkB signaling is essential for adaptive myelination, and methotrexate chemotherapy disrupts this signaling in a microglial activation-dependent manner [229]. More importantly, treatment with the TrkB partial agonist LM22A-4 rescued methotrexate-associated myelination deficits and alleviated cognitive deficits in mice [229]. These findings highlight the role of myelin dysfunction in chemotherapy-related cognitive impairment. However, whether pro-myelination compounds can prevent or alleviate cognitive impairment associated with chemotherapy remains to be further explored.

### COVID-associated neurological symptoms

COVID-19 survivors often experience long-term neurological complications, collectively referred to as post-COVID-19 syndrome or long COVID, which is characterized by fatigue, depression, anxiety, and memory issues [232]. Among these symptoms, cognitive impairment is frequently reported in clinical settings [233, 234]. A cohort study revealed a high incidence of cognitive impairment in COVID-19 survivors 12 months after discharge [235]. Furthermore, after adjusting for age, sex, education level, body mass index, and comorbidities, severe cases of COVID-19 were associated with a significantly higher risk of early-onset cognitive decline, while even nonsevere cases were linked to an elevated risk of early onset cognitive decline [235]. Similar findings have also been reported in other cohort studies [236–238].

Results from a recent study provided additional insights into the molecular basis of COVID-19-related cognitive impairment [26]. Besides reduced hippocampal neurogenesis, a decrease in the number of OLs in subcortical white matter was observed in mice infected with SARS-CoV-2, both weeks and months after infection. SARS-CoV-2 infection also led to reduced myelinated axons, resembling the effects seen in mice treated with chemotherapy agents [26]. Although relevant clinical evidence remains limited, a case report documented a COVID-19 survivor with sudden neurological impairment who exhibited new-onset multiple, non-enhancing demyelinating lesions in both the brain and spine [239]. While the exact mechanisms by which COVID-19 leads to cognitive impairment are still unclear, these findings allow for the hypothesis that myelin dysfunction may contribute, given the critical role of myelin in memory and cognition. Since COVID-19 is likely to coexist with humans for the foreseeable future, further exploration of the causal relationship between myelin dysfunction and COVID-associated neurological symptoms represents a valuable area for future research. Moreover, investigating whether remyelination therapies could benefit COVID-19 survivors with cognitive symptoms is an intriguing direction for further study.

### The future of remyelination therapy in aging and brain disorders

**Fig. 4:**
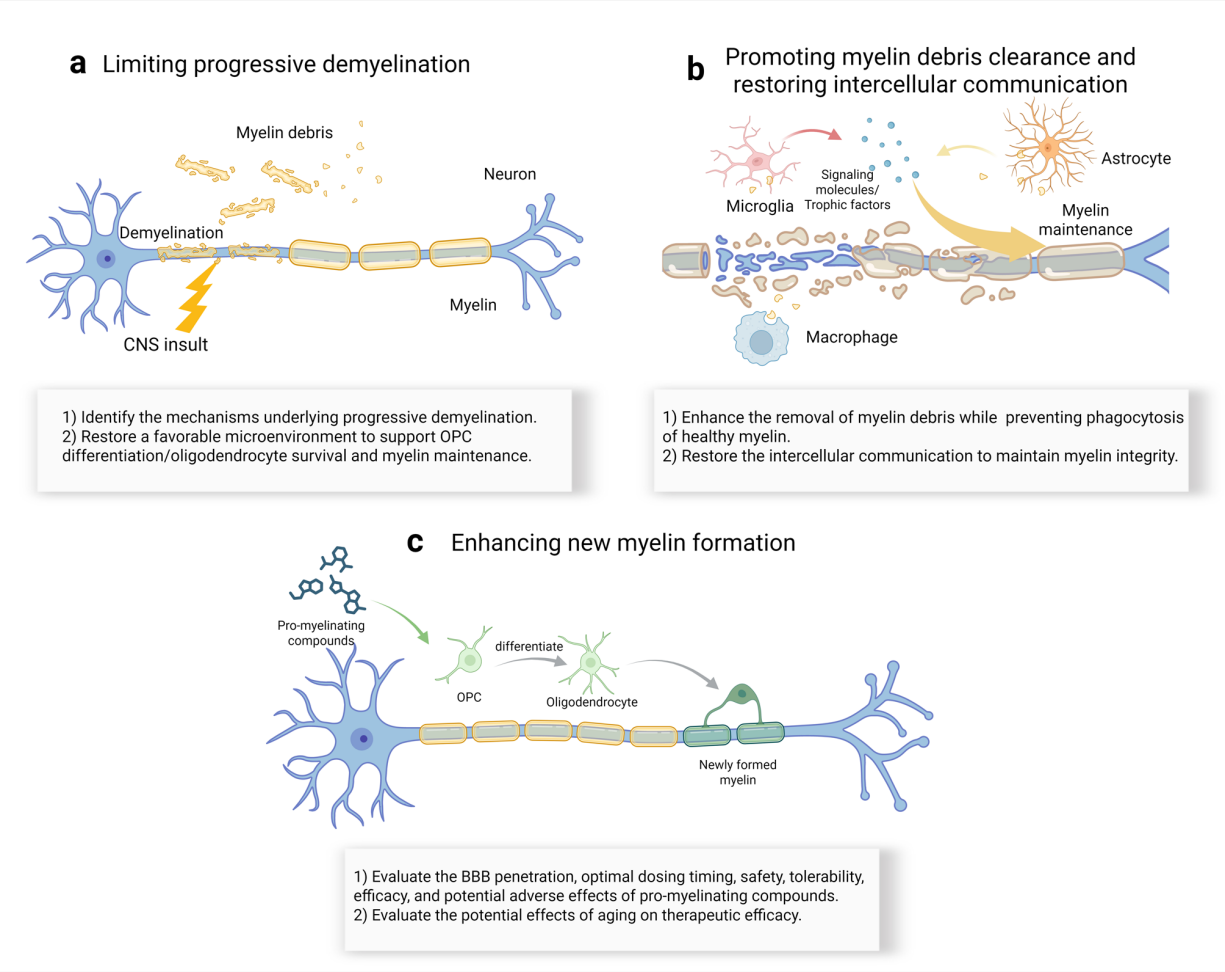
The future of remyelination therapy in aging and brain disorders. The framework for successful remyelination therapy includes: **a** limiting the progressive demyelination, **b** promoting myelin debris clearance and restoring intracellular communication, and **c** enhancing new myelin formation

The discovery of novel pro-myelinating compounds has led to promising therapeutic approaches for mitigating neurological deficits linked to myelin dysfunction. Nevertheless, relying on these pro-myelinating compounds alone may not be sufficient to yield translatable benefits unless the pathological alterations that contribute to ongoing demyelination and the unfavorable microenvironments for myelin maintenance are addressed. The successful development of remyelination strategies requires careful consideration of the multifaceted factors influencing myelin physiology. In the following section, we summarize and discuss the challenges and future perspectives for remyelination therapies (Fig. 4).

### Limiting progressive demyelination

Although remyelination therapies have shown promise in various demyelinating conditions, whether progressive demyelination or failure of remyelination is predominant in different conditions, or whether both can present, remains incompletely understood. For conditions characterized by progressive demyelination, addressing the ongoing demyelination would be the priority. In the case of MS, immunomodulatory strategies remain an essential part of therapeutic frameworks, as newly formed myelin can still be targeted and degraded by immune cells. Practically, combining immunomodulatory therapies with regenerative treatments has been proposed as a potential paradigm for future MS management [131, 145].

Similarly, the aging brain is characterized by both progressive myelin breakdown and remyelination failure, processes in which altered microenvironments appear to play a crucial role [94–96]. In this context, further investigation into the mechanisms driving microenvironment deterioration during aging, along with efforts to restore a favorable microenvironment, may pave the way for successful remyelination therapies for aging and aging-associated conditions. A therapeutic strategy that simultaneously limits ongoing demyelination while promoting remyelination is expected to achieve better outcomes.

Prolonged demyelination is also observed in other brain disorders, necessitating strategies that can limit its progression to achieve effective myelin repair. A deeper understanding of the mechanisms driving progressive demyelination across different pathological conditions, therefore, is required to enable precise and targeted interventions.

### Promoting Myelin debris clearance and restoring intercellular communication

The removal of myelin debris is a critical prerequisite for the successful myelin repair. Accumulation of myelin debris at lesion sites amplifies and perpetuates inflammatory responses, creating an obstacle to effective remyelination [240, 241]. Myelin debris clearance is predominantly mediated by microglia and macrophages [241, 242]. Upon CNS insults, macrophages are recruited to injury sites, where they play a critical role in clearing cellular debris, including lipid-rich myelin debris [242, 243]. The activation of TREM2, a protein essential for microglial phagocytic activity, has been shown to increase microglia-mediated phagocytosis of myelin debris, leading to a rise in OPCs, mature oligodendrocytes, restored myelin, and preserved axonal integrity in areas of demyelination [244]. Conversely, inefficient clearance of myelin debris by microglia impedes proper remyelination and results in aberrant myelin patterns within demyelinated lesion areas [245].

Astrocytes also play a critical role in the phagocytosis of myelin debris in various demyelinating CNS diseases. Myelin debris-containing astrocytes have been observed in autopsy tissues from patients with MS, ischemic stroke, and other demyelinating disorders [246]. Ablation of astrocytes impairs microglial recruitment to demyelinated areas, leading to delayed myelin debris clearance, hindered OPC differentiation, and failed remyelination [247]. By contrast, excessive or aberrant phagocytic activity of these cells could lead to the phagocytosis of healthy myelin, exacerbating demyelination [181, 248]. This highlights the importance of maintaining proper phagocytic activity—promoting effective debris removal while preventing immune attacks on healthy myelin.

In addition to their phagocytic role, astrocytes and microglia contribute essential signals for oligodendrocyte physiological processes. Astrocyte-derived BDNF, a key neurotrophin, supports myelin protein synthesis in demyelinated regions [249]. Microglia have recently been shown to regulate CNS myelin growth and integrity via the TGFβ1–TGFβR1 axis in microglia–oligodendrocyte communication, as well as through other signaling pathways [250, 251]. The close interaction between OLs, microglia, and astrocytes suggests that successful remyelination therapies should go beyond targeting OPCs and OLs. Other cell types involved in the processes of demyelination and remyelination could also be potential therapeutic targets.

### Enhancing new Myelin formation

Although the development of high-throughput platforms has facilitated the identification of numerous pro-myelinating compounds, and despite encouraging preliminary results, only a few of these compounds have progressed to clinical trials. Among the recently identified compounds, clemastine fumarate is the only agent tested in a phase II clinical trial involving patients with MS, with primary outcomes reported [148]. As one of the most extensively studied pro-myelinating compounds in experimental models, clemastine fumarate has been shown to effectively enhance myelination and promote functional recovery across various disease models, including aging, AD, depression, TBI, stroke, and hypoxic brain injury. However, recent studies have reported potential adverse effects associated with this ‘star’ drug [252, 253]. In neonatal mice, clemastine fumarate treatment during development (postnatal day 5 to day 20) led to a reduced number of nodes of Ranvier and myelinated axons in the corpus callosum, accompanied by decreased action potential conduction [252]. This effect may be attributed to clemastine’s antagonism of the M1 muscarinic acetylcholine receptor in other cell types. Microglia, which express the M1 muscarinic acetylcholine receptor, exhibited altered morphological complexity and ramification [252]. Additionally, CD11c^+^ microglial cells—a transient microglial subtype involved in myelinogenesis during development—were also reduced when clemastine was administered during this period [252]. A recent study investigated the effects of various clemastine fumarate treatment regimens in a lysolecithin-induced rabbit demyelination model [253]. The findings revealed that clemastine increased the number of mature oligodendrocytes at the expense of progenitor pool exhaustion. Additionally, short-duration dosing regimens—initiated either on the day of lysolecithin injection or five weeks post-injection and lasting for three weeks—were insufficient to increase the number of mature oligodendrocytes when compared to continuous treatment (starting on the day of lysolecithin injection and last eight weeks). Furthermore, late clemastine treatment (initiated five weeks after the induction of focal demyelination for three weeks) resulted in pronounced senescence in OPCs and DNA damage in mature oligodendrocytes [253]. This raises the possibility that enhancing OPC differentiation at different stages of demyelination may lead to distinct OL fates, which have not been fully characterized in most experimental models. In this context, results from preclinical studies should be interpreted cautiously, as they may overlook the potential side effects of these compounds despite their robust effects on myelination. Similar concerns arise with other pro-myelinating agents, as most target M1/M3 muscarinic acetylcholine receptors to promote OPC differentiation. However, these receptors are also expressed in other CNS cell types [254], making it challenging to exclude their effects on non-oligodendrocyte populations. A better understanding of these potential effects, and the extent to which they influence oligodendrocyte physiological processes, is crucial for developing more effective and reliable treatment regimens.

The blood-brain barrier (BBB) presents a major obstacle in the treatment of brain disorders. Therefore, evaluating the BBB permeability of identified pro-myelinating compounds is a crucial prerequisite for successful treatment. Nevertheless, the BBB permeability of these compounds has not been well characterized. To date, only benztropine, clemastine, solifenacin, miconazole, clobetasol, ESI1, and PIPE-307 have clearly demonstrated the ability to cross the BBB effectively. However, the brain bioavailability of these compounds remains incompletely understood. Comprehensive profiling of these parameters will be essential for determining therapeutic dosages and ensuring safe clinical application. Indeed, numerous physiological factors can limit the brain bioavailability of drugs. To address these challenges, various strategies have been proposed, including BBB penetration via passive transcytosis, intranasal administration, nanomaterial-based drug delivery systems, and light- or focused-ultrasound-mediated BBB opening [255–257]. Notably, recent studies have shown that the BBB exhibits circadian rhythms, with the highest efflux of xenobiotics occurring during the active period and the lowest during the resting period in both Drosophila and mice [258–260]. In a Drosophila seizure model, the anti-epileptic drug phenytoin was most effective when administered during the resting period compared to administration during the active period [258]. These findings may help explain the heterogeneous outcomes of drug treatments for brain disorders and underscore the importance of considering circadian regulation of the BBB to optimize therapies. Incorporating this property into therapeutic strategies is expected to enhance drug delivery to the brain and maximize its availability. In this context, exploring whether enhanced BBB penetration or brain retention can improve bioavailability and boost the therapeutic efficacy of pro-myelinating agents would be highly intriguing.

The timing of dosing for pro-myelinating compounds is also critical, as progressive diseases and acute CNS insults may require distinct treatment regimens. In TBI, initial priorities include managing hypoxia, hypotension, hypothermia, extracranial bleeding, intracranial pressure monitoring, airway management, and ventilation [261, 262]. Similarly, the acute management of ischemic stroke focuses on stabilizing the airway, breathing, and circulation; and restoring cerebral perfusion through intravenous thrombolysis or intra-arterial thrombectomy [178, 263]. For intracerebral hemorrhage, interventions such as anticoagulation reversal, external ventricular drainage, or neurosurgical removal of large cerebellar hematomas are the priority [178]. Remyelination therapy is unlikely to be considered until these critical acute events are managed.

Age is another key consideration for successful remyelination therapies. The aging brain exhibits prominent alterations in OPCs/OLs—including epigenetic modifications, microenvironment changes, and transcriptomic alterations, as described previously and reviewed in detail elsewhere [264]. These age-related changes in OPCs/OLs typically lead to diminished remyelination capacity, potentially compromising therapeutic efficacy [264]. This is supported by the evidence that OPCs isolated from aged rats exhibit a compromised inherent capacity for differentiation and are less responsive to the pro-differentiation signals compared to the OPCs from the young rats [265]. In diseases where age is a risk factor, aging-associated OPC/OL alterations may interact with disease-specific pathologies, potentially leading to exacerbated pathological consequences that impair remyelination and/or worsen myelin breakdown. In this case, rejuvenating the differentiation capacity of OPCs may be a priority. This might also be particularly relevant for diseases that are more prevalent in the elderly, such as ischemic stroke, where therapeutic responses could differ between young and aged individuals due to age-related declines in remyelination capacity. Thus, considering age as a variable may provide a more accurate assessment of the efficacy of remyelination therapies in aging-associated conditions.

Several clinical trials on pro-myelinating compounds are currently recruiting or ongoing.

These include a trial evaluating the efficacy of clemastine in MS patients with chronic demyelinated optic neuropathy (NCT02521311), a trial investigating the effects of combining clemastine and aerobic exercise on cognitive dysfunction in patients with schizophrenia (NCT06315972), a trial examining the effects of clemastine on improving white matter and enhancing standard antidepressant treatment in older adults with depression (NCT06591091), and a study assessing the effects of bazedoxifene on white matter myelin and functional outcomes in MS patients (NCT04002934). The results from these trials are anticipated to provide valuable insights into the safety, tolerability, efficacy, and potential adverse effects of newly identified pro-myelinating agents, potentially advancing these therapies toward clinical application. In addition to the neurological conditions discussed, other disorders, such as acute disseminated encephalomyelitis [266] and central pontine myelinolysis [267], are also characterized by demyelinating lesions in the brain. Emerging evidence further suggests that diabetes mellitus can lead to neurological symptoms and CNS myelin abnormalities [268–270]. Investigating whether remyelination therapies could improve neurological deficits associated with these conditions would be an area of great interest for further study.

## Conclusion

Beyond its role as an insulating sheath around axons, additional functional significance of myelin has come to light. Intact myelin not only provides metabolic support for axonal integrity but also plays a crucial role in various aspects of cognitive functioning. Compelling evidence suggests that adaptive myelination is essential for memory consolidation and motor learning, leading to considerable interest in this field. Importantly, myelin dysfunction is increasingly recognized as one of the core pathological features in aging-associated cognitive decline and various brain disorders. Enhancing myelination has shown potential in mitigating the neuropathology of these conditions. A deeper understanding of the mechanisms driving myelin dysfunction across different conditions, alongside further studies on the safety and efficacy of pro-myelinating compounds, holds potential for developing targeted therapies.

## Abbreviations

AD: Alzheimer’s disease
Aβ: Amyloid-beta
BBB: Blood-brain barrier
CSF: Cerebrospinal fluid
MS: Multiple sclerosis
TBI: Traumatic brain injury
mPFC: Medial prefrontal cortex
GLUT1: Glucose transporter 1
MCT: Monocarboxylate transporter
Myrf: Myelin regulatory factor
OL: Oligodendrocyte lineage cell
OPC: Oligodendrocyte precursor cell
GPER: G protein-coupled estrogen receptor 1
TLR7: Toll-like receptor 7
EAE: Experimental autoimmune encephalomyelitis
EphA4: Ephrin A4 receptor
EBP: Emopamil-binding protein
LDLR: Low-density lipoprotein receptor
LRP1: Low-density lipoprotein receptor-related protein 1
KOR: κ-opioid receptor
